# Biphasic Bone Implants through Hybrid Extrusion Printing of Thermoplastic Poly(lactic-*co*-glycolic) acid and Strontium-Modified Calcium Phosphate Bone Cement

**DOI:** 10.1021/acsomega.5c12496

**Published:** 2026-04-30

**Authors:** Max von Witzleben, Tilman Ahlfeld, Richard Frank Richter, Anna-Maria Placht, Constantin Greil, Corina Vater, Andreas Hoess, Sascha Heinemann, Stefanie Grom, Tatjana Fecht, Jan Marc Schehl, Tobias Wolfram, Frank Reinauer, Christian Bräuer, Günter Lauer, Michael Gelinsky, Anja Lode

**Affiliations:** † Centre for Translational Bone, Joint and Soft Tissue Research, 9169 University Hospital Carl Gustav Carus and Faculty of Medicine at Technische Universität Dresden, Fetscherstr. 74, Dresden 01307, Germany; ‡ INNOTERE GmbH, Meissner Str. 191, Radebeul 01445, Germany; § KLS Martin SE & Co. KG, Kolbinger Str. 10, Mühlheim 78570, Germany; ∥ Department of Oral and Maxillofacial Surgery, University Hospital Carl Gustav Carus at Technische Universität Dresden, Fetscherstr. 74, Dresden 01307, Germany

## Abstract

Biphasic scaffolds that integrate bioactive ceramics with biodegradable polymers hold considerable potential for bone regeneration, but they present challenges in terms of interfacial integration and processability. In this study, biphasic scaffolds consisting of alternating strands of strontium-modified calcium phosphate cement (SrCPC) and poly­(l-lactide-*co*-glycolide) (PLLA–PGA) were successfully fabricated using an optimized extrusion-based 3D printing and postprocessing protocol. Degradation studies in water showed that the presence of the SrCPC phase in the biphasic scaffolds buffered the acidic degradation products of PLLA–PGA, delaying and attenuating the pH drop over time and influencing the degradation of the polymer phase. Uniaxial compression tests revealed intermediate mechanical properties of the biphasic compared to monophasic PLLA–PGA and SrCPC scaffolds, which declined over the 24-week observation period. Aging experiments in cell culture medium under near-physiological conditions indicated a significant mutual influence of the two materials, as evidenced by an altered strontium and phosphate ion release profile from the SrCPC phase and the formation of a different surface structure on the materials in the biphasic system compared to the monophasic system. This was also reflected by the cellular response of osteoblast-like SaOS-2 cells, showing a local heterogeneity in cell–material interactions. Nevertheless, the cell experiments demonstrated the strong positive effect of the bioactive SrCPC component in the biphasic scaffolds on cell proliferation and alkaline phosphatase activity, which were significantly higher than those in the monophasic PLLA–PGA scaffolds.

## Graphical abstract

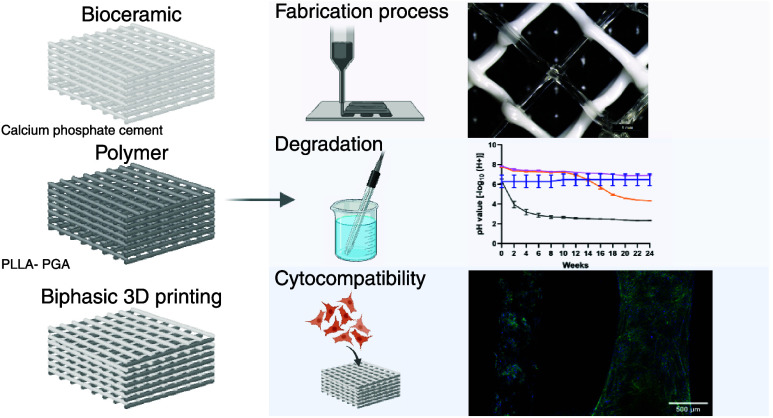

## 1 Introduction

Bone tissue has a high regenerative capacity that allows spontaneous healing of small defects in otherwise healthy patients. However, large bone defects, resulting, for example, from tumor resection, trauma, or infection, must be treated by filling with grafts to reconstruct the defect and restore tissue function. Since the current gold standard, autologous bone, is limited in availability and requires a second surgical intervention at the donor site, much attention has been paid to the development of biodegradable biomaterials that temporarily fill the defect and support bone tissue regeneration.^1,2^

Biomaterials based on the polyester poly­(lactic acid) (PLA) are already in clinical application for bone regeneration and approved by the Food and Drug Administration (FDA), which are characterized by their high biocompatibility and favorable mechanical stiffness.^3–5^ The degradation rate of PLA can be tailored by adjusting its crystallinity.^6^ PLA exists in three isomeric forms: l-lactide, d-lactide, and racemic (d,l) lactide; for most applications, the (L) isomer (PLLA) is chosen because it is preferentially metabolized in the body.^4^ The degradation mechanism of polyesters is based on the random hydrolysis of their ester bonds, releasing their monomers, such as lactic acid in the case of PLA. PLA is more hydrophobic than the polyester poly­(glycolic acid) (PGA) and is, therefore, more resistant to hydrolytic attack than PGA.^4^ Copolymers of poly­(lactic-*co*-glycolic acid) (PLGA) allow for a much better control over the degradation rate compared with its constituent homopolymers, PLA and PGA.^5^ As thermoplastics that melt at elevated temperatures and solidify again on cooling, PLA-based biomaterials are applicable for 3D printing, for instance, by fused filament fabrication (FFF), also known as fused deposition modeling (FDM) or filament freeform fabrication.^7^ Thus, 3D scaffolds and implants with a defined pore structure as well as defect-specific shape and size can be fabricated.

Another class of biomaterials that is already in clinical application is calcium phosphate (CaP)-based ceramics. Prominent representatives include self-setting calcium phosphate cements (CPC) as injectable, FDA-approved bone replacement materials. CPC consist of a precursor powder of one or more CaP phases and a liquid phase; after mixing, dissolution and reprecipitation reactions occur that lead to cement hardening.^8^ CPC based on α-tricalcium phosphate (α-TCP), such as those used in this study, form nanocrystalline (calcium-deficient) hydroxyapatite with low solubility during cement setting.^8,9^ CPC are biodegradable and possess excellent osteoconductivity and biocompatibility.^10^ They can be modified with strontium ions to achieve local long-term delivery of Sr^2+^, which stimulate osteogenesis, resulting in increased bone regeneration.^11–13^ By suspending the α-TCP-based precursor powder in a biocompatible oil-based carrier liquid, storable CPC pastes have been generated for ready-to-use clinical applications; the cement setting reaction starts only in contact with aqueous solutions, when the oil is exchanged for water.^14,15^ These oil-based CPC pastes are also applicable for the extrusion-based 3D printing of scaffolds and implants with high shape fidelity.^16–18^ As the process conditions during printing of CPC are mild and the setting reaction does not cause heat development and has only a minor influence on ion concentrations and pH value in the surrounding environment, they have also been processed in combination with (cell-laden) hydrogels by multichannel extrusion printing to generate biphasic constructs, e.g., for the repair of osteochondral defects.^19–21^

Albeit already in clinical use, both material classes have advantages and limitations. Thermoplastic polyesters such as PLA or PLGA exhibit favorable mechanical stiffness, but their intermediate degradation products cause a local pH drop that, in turn, can induce an inflammatory reaction and damage bone cells.^22^ CaP-based materials, such as nanocrystalline CPC, are characterized by their high bioactivity and physicochemical proximity to natural bone minerals, but they are brittle and, therefore, often cannot withstand mechanical loads without breaking.^8,23^ Therefore, research is focused on the development of improved implant materials by the combination of the different materials. One common strategy is the generation of composites, which involves integrating CaP-based (nano/micro) particles as fillers into the polymer matrix.^24,25^ In our previous work, we fabricated composite scaffolds consisting of the copolymer PLLA–PGA, approved for specific clinical indications (FDA K121606), and different mineral fillers (calcium carbonate, strontium carbonate, strontium-modified hydroxyapatite (SrHAp), α-tricalcium phosphate (α-TCP), β-tricalcium phosphate (β-TCP)) by 3D printing and investigated their degradation behavior.^26^ The integration of the mineral filler particles (10 wt %) affected the printing properties of the polymer; however, by adjusting the process parameters, printing with high accuracy was still possible. During aging, the CaP phases (SrHAp, α-TCP, β-TCP) were able to buffer the acidic degradation products of PLLA–PGA. On the other hand, 10 wt % mineral filler particles did not seem to improve the bioactivity of PLLA–PGA scaffolds, but resulted in a (undesirable) moderate dimensional increase.^26^

In the present work, we explored another strategy to combine the polymer PLLA–PGA with a CaP phase. By using a multichannel/multitool 3D printing system, PLLA–PGA was coprinted with a strontium-modified CPC paste (SrCPC)^15^ to create biphasic scaffolds by alternating the deposition of the two materials. We systematically evaluated the printability of the individual and combined materials and characterized the resulting biphasic scaffolds *in vitro* by conducting degradation studies in water as well as in cell culture medium to simulate physiological conditions. We also investigated the cellular response of osteoblastic cells to the biphasic material system. We tested the hypotheses that the biphasic scaffolds would, in comparison to monophasic PLLA–PGA scaffolds, exhibit (i) a reduced pH drop during degradation due to the buffering effect of the mineral phase and (ii) improved cell colonization with osteogenically differentiated cells due to the bioactivity and osteoconductivity of the SrCPC component, as well as a significant release of osteostimulatory strontium ions. To elucidate the interaction between the two material phases and their environments, we supplemented our analysis with elemental surface characterization and ion exchange profiling over a period of 70 days.

## 2 Materials and Methods

### 2.1 Printing Materials

PLLA–PGA (weight ratio 85:15) granules were obtained from Evonik Industries AG (Essen, Germany). A strontium-modified, self-setting calcium phosphate cement paste (SrCPC), consisting of a precursor powder (α-tricalcium phosphate, dicalcium phosphate anhydrous, strontium carbonate, hydroxyapatite, and potassium hydrogen phosphate as a catalyst) mixed into an oil-based carrier liquid (Miglyol, Cremophor ELP, and Amphisol A)^15^ was manufactured by INNOTERE GmbH (Radebeul, Germany).

### 2.2 Scaffold Fabrication

Printing was conducted utilizing a BioScaffolder 3.1 (GeSiM mbH, Radeberg, Germany) equipped with nontempered pneumatic print heads for 3D printing of SrCPC. An externally heated cartridge with an additional nozzle for heating was applied to the print head for the FFF of PLLA–PGA. An overview of the relevant optimized fabrication parameters for monophasic and biphasic scaffolds is provided in Table 1. As a printing substrate for all scaffold types, a conventional glass plate was used. Extrusion of SrCPC was carried out using conical plastic needles (Globaco GmbH, Rödermark, Germany) with an inner diameter of 410 μm. After 3D printing of the monophasic SrCPC, the obtained scaffolds were incubated in a water-saturated atmosphere (>95% humidity) at 37 °C for 3 days for cement setting and hardening. The PLLA–PGA granulate was filled into the high-temperature cartridge and tempered at 170 °C for 2 h for optimal and homogeneous processing. During printing, the metal nozzle was heated to 180 °C to enhance the printing properties. For biphasic PLLA–PGA/SrCPC scaffolds, the printing procedure resembled that of monophasic scaffolds, but the printing speed of PLLA–PGA as well as the layer height of SrCPC was adjusted to guarantee optimal printing outcomes. After manufacturing, the biphasic scaffolds were incubated in a water-saturated atmosphere (>95% humidity) at 37 °C for 3 days, followed by intensive washing in 0.9% NaCl to remove the oil-based carrier liquid. For cell culture and degradation experiments, monophasic and biphasic scaffolds were sterilized using low-dose γ-irradiation with max. 25 kGy.

**Table 1:** Processing Parameters for the Extrusion 3D Printing of SrCPC and FFF of PLLA–PGA for Monophasic and Biphasic Scaffold Structures

	needle size [μm]	printing speed [mm·s^–1^]	pressure [kPa]	print head temperature [°C]	layer height [μm]
***monophasic scaffolds***					
SrCPC	410	10	120	room temp.	260
PLLA–PGA	400	4	550	170	320
***biphasic scaffolds***					
SrCPC	410	10–12	120	room temp.	270
PLLA–PGA	400	1.5	550	170	320

For the degradation studies (described in Sections 2.4 and 2.5), scaffolds with a PLLA–PGA contour, a height of 5 mm, and a 90°-lattice structure, composed of a strand distance of 1.5 mm, and a base area of 10 × 10 mm were printed using the printing parameters listed in Table 1. For the cell culture experiments (described in Section 2.7), scaffolds consisting of four layers were printed with a 90° interwoven lattice structure, a contour (biphasic with PLLA–PGA contour), and a round shape (Ø 13 mm) to fit into 24-well cell culture plates. The interwoven structure allowed for imaging the cell behavior not only on strands deeper within the scaffold architecture, but also the comparison of the cell behavior on different material phases in close proximity.

### 2.3 Characterization of Printing Fidelity

Filament fusion tests of SrCPC and PLLA–PGA were carried out according to Ribeiro et al.^27^ Six layers of strands were printed as meanders with increasing strand distances and a 90° layer-to-layer shift (Figure 1a,d). The printed structures were imaged with a stereo light microscope (Leica M205 C equipped with DFC295 camera, Germany). The strand widths, strand distances, and fused segment lengths were measured using ImageJ (1.51w, National Institutes of Health, USA). The shape fidelity ratio was determined by dividing the fused segment length (*f*_s_) by the strand width (*f*_d_) and assigning it to the allocated strand distance (d) (Figure 1g). The optimal shape fidelity was defined as a ratio of 1.

**Figure 1:**
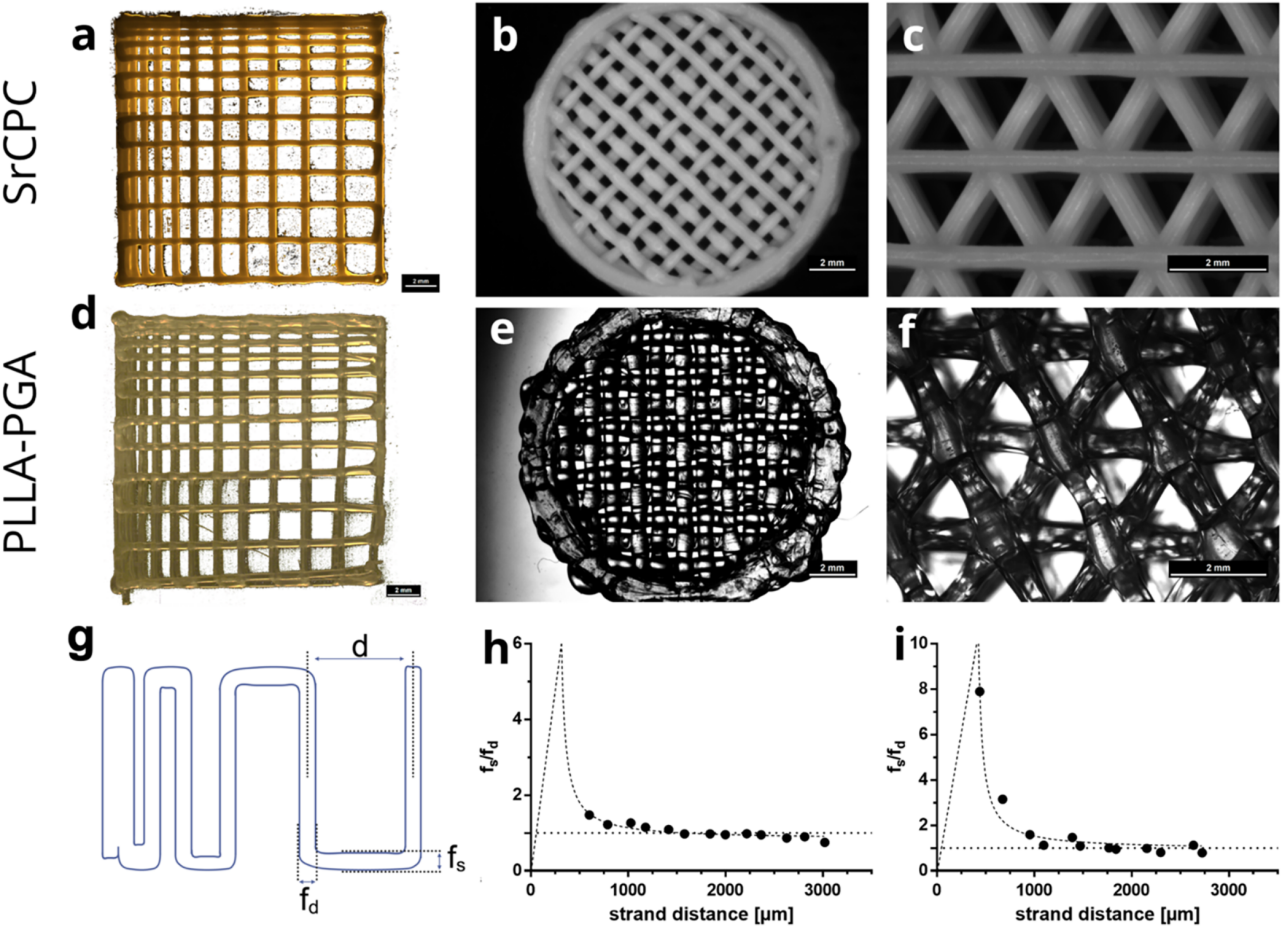
Monophasic 3D extrusion printing of SrCPC and fused filament fabrication of PLLA–PGA scaffolds with BioScaffolder 3.1. (a–c, e–g) Stereo light microscopic images of scaffolds with different pore structures. (a, d) Meandering pore structures with a 90° layer-to-layer shift. These resemble the filament fusion test for each layer, as sketched in (g). Created in BioRender (2026), https://BioRender.com/l5ae864. The respective shape fidelity ratio (*f*_s_/*f*_d_) is shown in (h, i).

### 2.4 Aging in Water and Analysis of Degradation

Monophasic PLLA–PGA and SrCPC scaffolds, as well as biphasic PLLA–PGA/SrCPC scaffolds, sterilized by γ-irradiation, were incubated in deionized water at 37 °C (4 scaffolds in 50 mL; in total, 20 scaffolds per group were analyzed) over 24 weeks, referring to DIN EN ISO 10993-13.^28^

#### 2.4.1 Measurements of pH Value and Conductivity

The pH value and conductivity in the incubation batches (*n* = 3) were monitored over the incubation period with a pH/conductivity meter (Multi 9260 IDS with pH electrode Sentix 980 IDS and conductivity electrode TetraCon 925; WTW, Xylem Analytics, Weilheim, Germany).

#### 2.4.2 Mechanical Testing

Uniaxial compression testing was performed on a universal testing machine (Zwick-Roell Z010, Zwick/Roell, Germany) equipped with a 20 kN load cell and operated in displacement control at a constant crosshead speed of 5 mm·min^–1^ (deformation rate) up to a standard displacement of 3.5  mm or specimen failure, whichever occurred first. Young’s modulus (*E*) was determined from the initial linear region of the engineering stress–strain curve by linear least-squares fitting. The compressive strength (σc) was defined as the peak (maximum) engineering stress prior to failure (local maximum of the stress–strain curve).

#### 2.4.3 Differential Scanning Calorimetry

Samples taken at weeks 0, 2,4, and 24 of incubation were desiccator-dried and analyzed by differential scanning calorimetry (DSC; 214 Polyma; Netzsch, Selb, Germany) in accordance with DIN EN ISO 11357-3.^29^ The samples were heated from −20 to 210 °C at a heating rate of 10 K/min (first heating), cooled down at a cooling rate of 10 K/min, and heated again from −20 to 210 °C at a heating rate of 10 K/min (second heating). The flow rate of the nitrogen gas was 60 mL/min. The masses of the respective material phases of the biphasic scaffolds were taken into account.

### 2.5 Aging in Cell Culture Medium and Analysis of Bioactivity

Monophasic SrCPC and PLLA–PGA scaffolds as well as biphasic PLLA–PGA/SrCPC scaffolds were incubated in 70% EtOH for 30 min for disinfection and then rinsed in cell culture medium consisting of Dulbecco’s Modified Eagle’s Medium (DMEM) supplemented with 15% fetal calf serum (FCS; Corning, USA), 100 U/mL penicillin, and 100 μg/mL streptomycin (PS) overnight to remove the residual carrier liquid and EtOH. After removal of the supernatant (time point: day 0), all samples were incubated in 1 mL of cell culture medium for over 70 days in an incubator (37 °C, 5% CO_2_, humid atmosphere). The medium was exchanged after 1, 3, 7, 9, 14, 21, and 28 days of incubation.

#### 2.5.1 Scanning Electron Microscopy and Energy-Dispersive X-ray Spectroscopy

After 70 days of incubation, samples were collected for analysis by scanning electron microscopy (SEM). The scaffolds were fixed with carbon slides on aluminum sample carriers before sputter coating with gold and imaged using a ZEISS DSM 950 equipped with a field emission gun (Carl Zeiss AG, Oberkochen, Germany). The microscope was operated in the SEM mode at a voltage of 4 kV (spot size of 3) with a field emission gun. For energy-dispersive X-ray spectroscopy (EDX), a voltage of 7 kV was necessary.

#### 2.5.2 Ion Release/Uptake

Supernatants collected during the 70-day incubation period were analyzed by inductively coupled plasma-optical emission spectrometry (ICP-OES, Plasma Quant PQ9000 Elite, Analytik Jena, Germany) to quantify the release/uptake of calcium, phosphate, and strontium ions, as described recently.^26^ The following emission lines were used for measurements: 315.887 nm (Ca), 213.618 nm (P), and 216.596 nm (Sr).

### 2.6 Cell Culture Experiments

#### 2.6.1 Seeding and Cultivation

The osteoblast-like cell line SaOS-2 (ATCC 243; DSMZ, Braunschweig, Germany) was used to study the cell response to biphasic scaffolds in a direct contact culture. The cells were expanded in a cell culture medium at 37 °C and 5% CO_2_. Scaffolds were preincubated in cell culture medium overnight and seeded with cells at different densities (5 × 10^4^ or 5 × 10^5^ cells per scaffold) in 24-well cell culture plates. After initial cell attachment within 24 h of incubation at 37 °C and 5% CO_2_, the seeded scaffolds were transferred to fresh 24-well plates, and 1 mL of cell culture medium containing the osteogenic supplements, 10^–7^ M dexamethasone, 50 μM ascorbic acid-2-phosphate, and 10 mM β-glycerophosphate (all from Sigma-Aldrich, Germany), was added to each scaffold. During cultivation, the medium was changed twice a week. Samples were collected for biochemical analysis of cell number and alkaline phosphatase (ALP) activity as well as for microscopic analysis of cell adhesion, morphology, and density after 1, 7, 14, and 21 days of culture.

#### 2.6.2 Quantification of Cell Number and ALP Activity

Cell-seeded scaffolds, taken at different time points of culture, were washed with phosphate-buffered saline (PBS; ThermoFisher, USA) and stored at −80 °C until biochemical analysis, thawed, and incubated in 500 μL of lysis buffer (1% Triton X-100 in PBS) for 50 min on ice. Lactate dehydrogenase (LDH) activity was determined in the lysates using the CytoTox 96 Non-Radioactive Cytotoxicity Assay (Promega, USA) according to the manufacturer’s instructions by measuring the absorbance at 490 nm (Infinite M200 Pro, Tecan, Switzerland) and correlated with the cell number using a calibration line from the LDH activity of defined cell numbers. The activity of alkaline phosphatase (ALP) was determined by incubation of aliquots (20 μL) of the same cell lysates used for cell number determination with 80 μL ALP substrate solution (1 mg/mL p-nitrophenylphosphate in 0.1 M diethanolamine, 0.1% Triton X-100, 1 mM MgCl_2_, pH 9.8; all from Sigma-Aldrich) at 37 °C. After 10 min, the enzymatic reaction was stopped by adding 1 M NaOH, and the absorbance was read at 405 nm (Infinite M200 Pro). Specific ALP activity was calculated by correlating the absorbance to a p-nitrophenol calibration line and the respective cell numbers.

#### 2.6.3 Fluorescence Microscopy

Cell-seeded scaffolds were collected at different time points of culture, washed with PBS, and fixed in 4% formaldehyde in PBS. After incubation in 0.2% Triton X-100 to permeabilize the cells, the samples were incubated in 1% bovine serum albumin (BSA) in PBS to minimize unspecific staining and thereafter stained with DAPI (Sigma-Aldrich) and Alexa Fluor 488 phalloidin (Invitrogen) to visualize the cell nuclei and actin cytoskeletons. Imaging of the stained samples was performed by using a Keyence AIO Fluorescence Microscope BZ-X800 (Keyence, Japan).

### 2.7 Statistical Analysis

All values were evaluated using one-way Analysis of Variance (ANOVA), followed by Tukey’s multiple comparison test with GraphPad Prism 9 software. Significant differences were assumed at *p* < 0.05.

## 3 Results

### 3.1 Fabrication of Biphasic Scaffolds of SrCPC and PLLA–PGA

As a starting point, the shape fidelity of monophasic 3D printed scaffolds consisting of either SrCPC or PLLA–PGA was evaluated (Figure 1). The parameters for monophasic printing were determined to obtain optimally shaped scaffolds. Both SrCPC and PLLA–PGA could be extruded with the BioScaffolder 3.1 using needles with inner diameters of 410 and 400 μm, respectively. Although the SrCPC paste could be processed as delivered, the PLLA–PGA pellets were melted in a high-temperature extrusion head before extrusion. To investigate the smallest printable pore size, a multiplayer pore size structure was developed based on a filament fusion test with a 90° shift between meandering strands in each layer (Figure 1a,d). There was a tendency that the smallest pore sizes were closed for PLLA–PGA but not for SrCPC. Nevertheless, the high concurrence of the design and printed construct proves a high shape fidelity for both monophasic materials. Additionally, other scaffold architectures were tested: small scaffolds with a diameter of 13 mm, a layer orientation of 90°, and an interwoven strand arrangement with a strand distance of 1 mm could be successfully fabricated without distinct failures of the printed shape, irrespective of the used material (Figure 1b,f). Also, scaffolds printed with layer orientations of 60° (Figure 1c,g) could be printed successfully. Subsequently, a quantitative evaluation of the printing fidelity of the scaffolds shown in Figure 1a,d, according to Ribeiro et al.,^27^ evidenced the general good printability of both SrCPC and PLLA–PGA (Figure 1g–i). For PLLA–PGA at pore sizes below 1 mm, the fidelity ratio *f*_s_/*f*_d_ was considerably higher than 1, but at pore sizes of approximately 1 mm, the ratio reached an optimum value of 1. For SrCPCs with pore sizes below 1 mm, fidelity ratios close to 1 were detected, which were within an acceptable deviation from the optimal ratio of 1. Consequently, biphasic scaffolds were printed with pore sizes of at least 1 mm to ensure high shape fidelity.

Figure 2 shows the scaffold designs (a, e, and i) and the corresponding printing results obtained for a multilayer architecture (15 layers) and scaffold edge length of 13 mm with a wood-stack architecture for both monophasic and biphasic scaffolds. In the fabrication of biphasic scaffolds, material-specific printing parameters required adjustment to accommodate differences in processing behavior. Specifically, the printing velocity of PLLA–PGA was reduced relative to that of the monophasic constructs to promote adequate bonding at the material interfaces. Interlayer adhesion is a well-known challenge in multimaterial 3D printing, particularly for dissimilar phases. It was found that incorporating a peripheral PLLA–PGA contour around the scaffold improved the overall structural integrity. In contrast, a SrCPC contour was not feasible, as the attachment of the PLLA–PGA was too low for reliable strand placement. Similarly, SrCPC deposited onto PLLA–PGA exhibited weaker strand attachment compared to the SrCPC-on-SrCPC contact, resulting in a reduction in strand linearity and local printing accuracy (arrows in Figure 2j,k). Despite these deviations in shape fidelity compared to monophasic constructs, successful biphasic printing of SrCPC/PLLA–PGA scaffolds was achieved.

**Figure 2:**
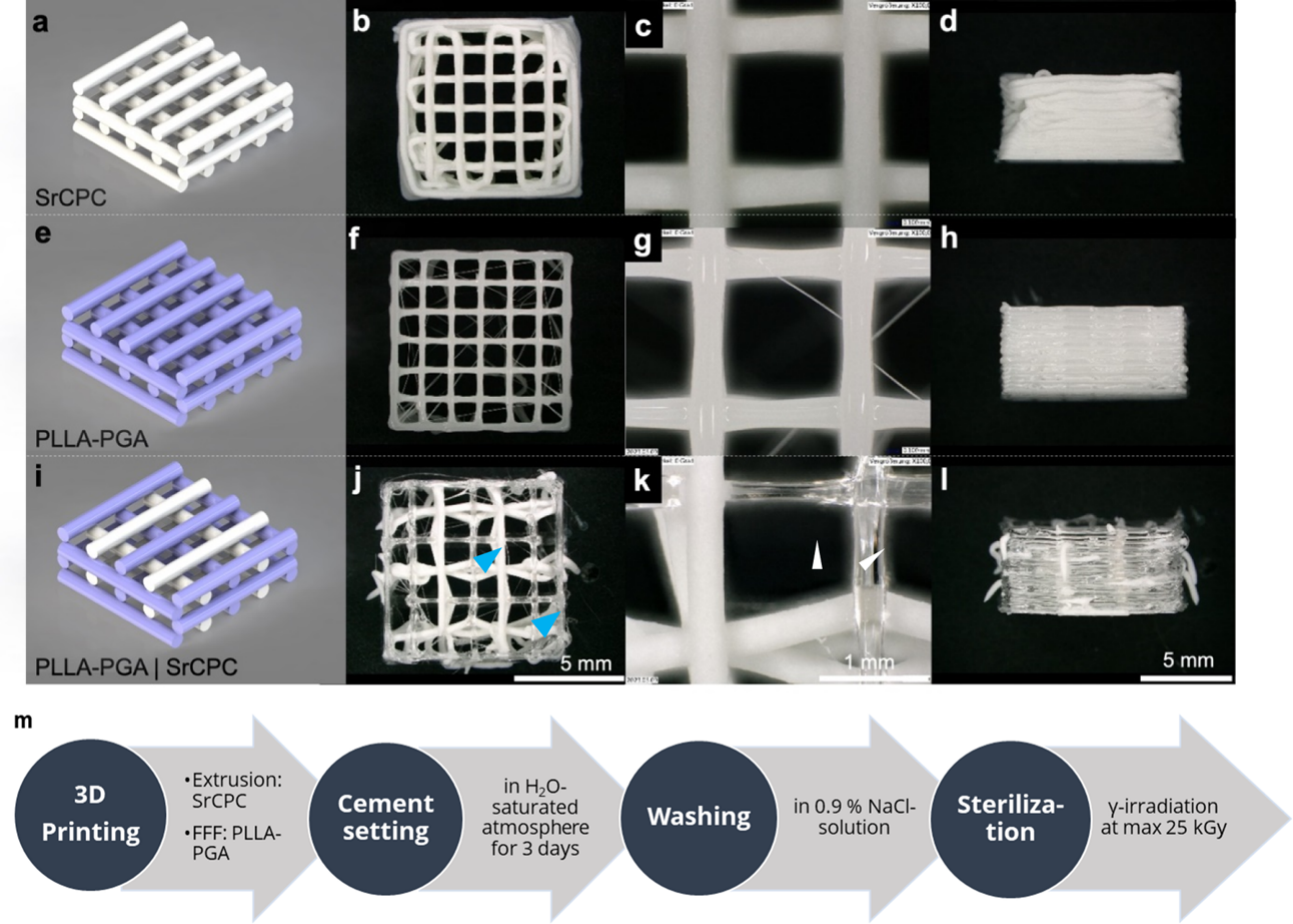
Monophasic and biphasic printing of PLLA–PGA/SrCPC scaffolds. Schematic presentation of the three scaffold designs (a, e, i) and stereo microscopic images of the printed scaffolds in a wood-stack architecture ((b, f, j): top view; (c, g, k): top view, detail; (d, h, l): side view). The blue arrows indicate that the SrCPC attachment is weaker on the PLLA–PGA strands than on the SrCPC-on-SrCPC contacts. Schematic presentation of the whole fabrication procedure, including postprocessing (m).

A crucial aspect of the biphasic material system is the postprocessing of printed scaffolds. Monophasic PLLA–PGA scaffolds must cool to solidify. For cementous scaffolds like SrCPC, it has been shown previously that the optimal solidification procedure is a setting process in a water-saturated atmosphere,^30^ leading to the formation of nanocrystalline strontium-substituted hydroxyapatite.^15^ Following initial setting under humid conditions for 3–5 days, the removal of the oil-based carrier liquid from (Sr)­CPC scaffolds is typically performed using organic solvents such as acetone.^31^ However, this approach is not suitable for biphasic PLLA–PGA/SrCPC scaffolds, as acetone degrades the thermoplastic phase by inducing polymer dissolution. Based on these considerations, a postprocessing protocol was developed, as illustrated in Figure 2m. Immediately after printing, the scaffolds were transferred to a humidified chamber with a water-saturated atmosphere at 37 °C, facilitating the initial SrCPC setting. After 3 days of incubation, the scaffolds were carefully detached from the substrate and intensively rinsed with 0.9% NaCl solution to remove residual carrier liquid from the cement surfaces. Finally, the samples were vacuum-dried for storage and sterilized using low-dose γ-irradiation (max. 25 kGy) to preserve the structural and mechanical integrity of the polymer phase.

### 3.2 Degradation Study in Water Including Mechanical Tests

Following the guidelines of DIN EN ISO 10993-13:2010-11, we conducted a degradation study in deionized water and evaluated the mechanical properties over a 24-week period. The pH value and conductivity of the supernatants were measured every 2 weeks (Figure 3a,b). The measurement of the pH revealed a gradual acidification in all PLLA–PGA-containing scaffold types, indicative of polymer degradation. In monophasic PLLA–PGA scaffolds, an immediate and rapid drop in pH was observed, which remained stable at ∼ 2.5 after 6 weeks. In contrast, the supernatants of monophasic SrCPC maintained a physiological pH of approximately 7.5–8 throughout the study. Biphasic scaffolds preserved a physiological pH until week 12, followed by a decrease to ∼4.5, where it stabilized.

**Figure 3:**
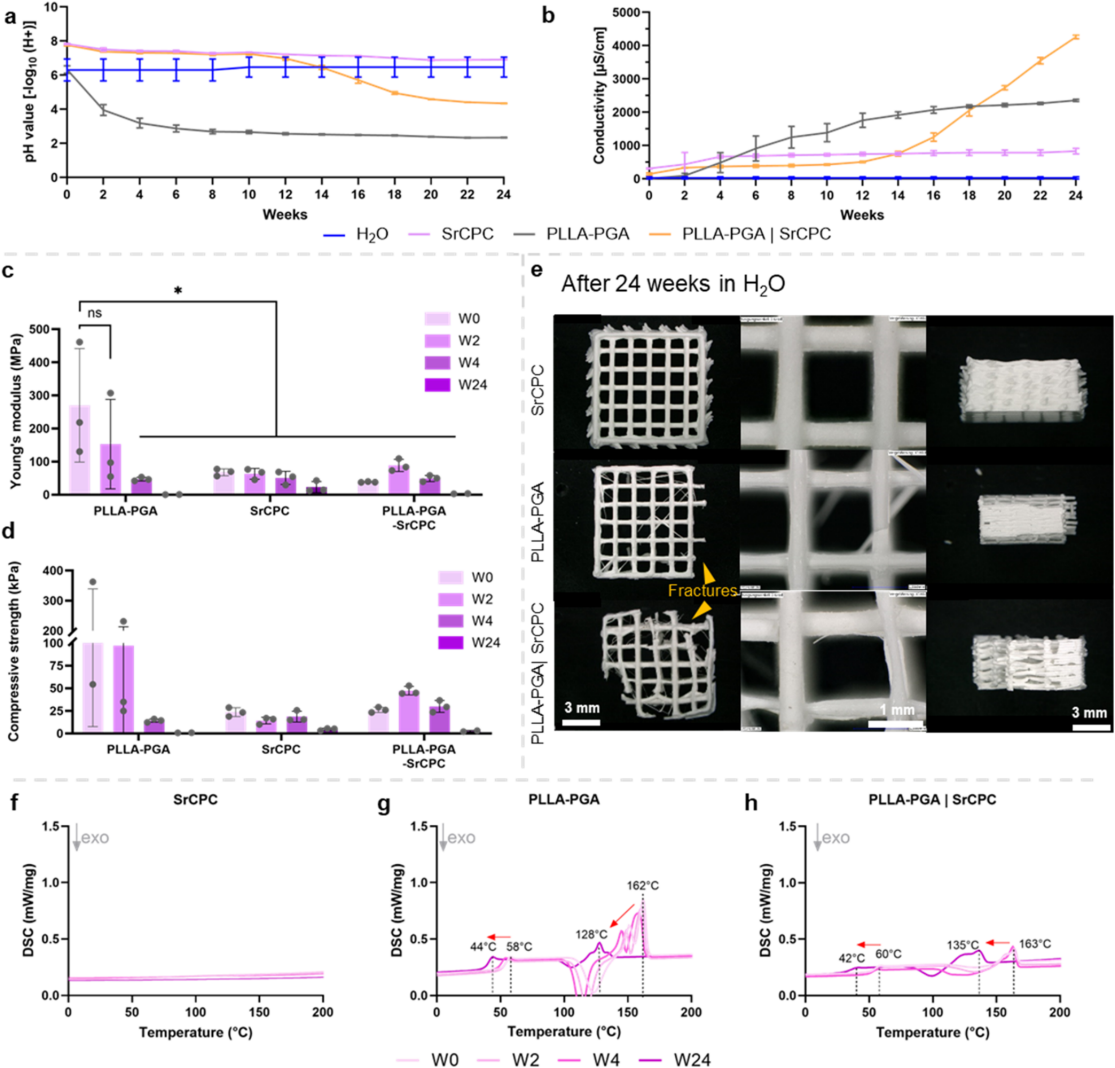
Characterization of the scaffold properties over the course of 24 weeks of incubation in deionized water: measurement of the pH value (a) and conductivity (b) (W0–4: *n* = 3, W24: *n* = 2 measurements); comparison of the mechanical properties by determining Young’s modulus ((c); *p* < 0.05, ns= nonsignificant) and compressive strength (d) (*n* = 3); microscopic images of the scaffolds after 24 weeks (e); and DSC data of all scaffold compositions ((f–h), *n* = 2).

The conductivity measurements supported these findings. In monophasic scaffolds, the conductivity increased in PLLA–PGA samples, suggesting the continuous formation of electrically charged hydronium ions, thus reflecting ongoing polymer degradation; whereas SrCPC showed a constant conductivity, indicating steady ion release. Biphasic scaffolds exhibited consistently lower conductivity compared to monophasic SrCPC up to week 12, consistent with their reduced SrCPC content. Parallel to the decrease of pH after week 12, the conductivity increased strongly, and at week 24, the conductivity of the biphasic scaffolds more than doubled relative to that of the monophasic PLLA–PGA. This supports the pH data and suggests a significant increase in ion release, thereby indicating strong degradation behavior.

To further assess degradation, uniaxial compressive tests were performed on scaffolds retrieved at weeks 0, 2, 4, and 24 to determine the Young’s modulus and compressive strength (Figure 3c,d). Monophasic PLLA–PGA scaffolds showed a marked decline in both Young’s modulus and compressive strength over time, whereas monophasic SrCPC maintained comparable mechanical properties for up to 4 weeks. A noticeable reduction in the mechanical stability of the SrCPC scaffolds was only observed at week 24. Biphasic scaffolds exhibited intermediate mechanical behavior compared with monophasic samples. Up to week 4, they displayed similar Young’s modulus and slightly enhanced compressive strength relative to pure SrCPC. However, by week 24, both values decreased to levels comparable with those of the PLLA–PGA scaffolds. Microscopic images taken after 24 weeks of incubation (Figure 3e) further support these findings: monophasic SrCPC scaffolds retained full structural integrity, while PLLA–PGA scaffolds showed partial disintegration. The biphasic scaffolds exhibited partial fractures and loss of larger fragments. Additionally, distinct whitening of the PLLA–PGA strands was observed, further indicating advanced degradation as a result of structural changes in the polymer matrix.

Differential scanning calorimetry at 10 K/min was employed to monitor the thermal behavior of the various scaffold constructs (Figure 3f–h). As anticipated, the monophasic SrCPC maintained stable thermal properties at the temperatures used throughout the observation period, indicating negligible material modification over time. In contrast, the monophasic PLLA–PGA scaffold exhibited a marked decrease in the glass transition temperature (*T*_g_) and melting temperature (*T*_m_): *T*_g_ decreased from 58 to 44 °C, and *T*_m_ decreased from 162 to 128 °C. Interestingly, two endothermic melting peaks were visible within the first 4 weeks, which then merged into one after 24 weeks. Furthermore, the typical exothermic peaks between 100 and 130 °C known for PLLA–PGA blends^32^ were visible. A comparable trend was observed for the biphasic PLLA–PGA/SrCPC scaffolds, with the *T*_g_ and *T*_m_ shifting from 60 to 42 °C and from 163 to 135 °C, respectively. Interestingly, the exothermic peaks were significantly less pronounced in biphasic conditions.

### 3.3 Aging in Cell Culture Medium

Although scaffold degradation in H_2_O provides useful insights into the effect of SrCPC on the degradation of PLLA–PGA, it lacks physiological relevance, as human blood contains a wide range of ions and proteins that modulate this process. Similar to our previous work,^26^ an additional 70-day degradation study was conducted with samples immersed in the cell culture medium to study the interaction of both materials in an *in vivo*-like environment. As observed in H_2_O, PLLA–PGA strands in both monophasic and biphasic scaffolds turned whitish after several weeks, due to a change in light scattering caused by microcrack and pit formation,^33^ indicating ongoing degradation in the medium (Figure 4). SEM analysis revealed further surface changes after 70 days: Monophasic SrCPC scaffolds retained their characteristic apatitic nanocrystalline surface morphology, as previously described,^18^ whereas sediment deposition was observed on the monophasic PLLA–PGA scaffolds, similar to our previous observations.^26^ In biphasic scaffolds, the SrCPC surface exhibited a more rounded appearance with flat patches—suggesting surface accumulation, while the PLLA–PGA strands displayed numerous small pores and the absence of sediments, as observed in the monophasic scaffolds.

**Figure 4:**
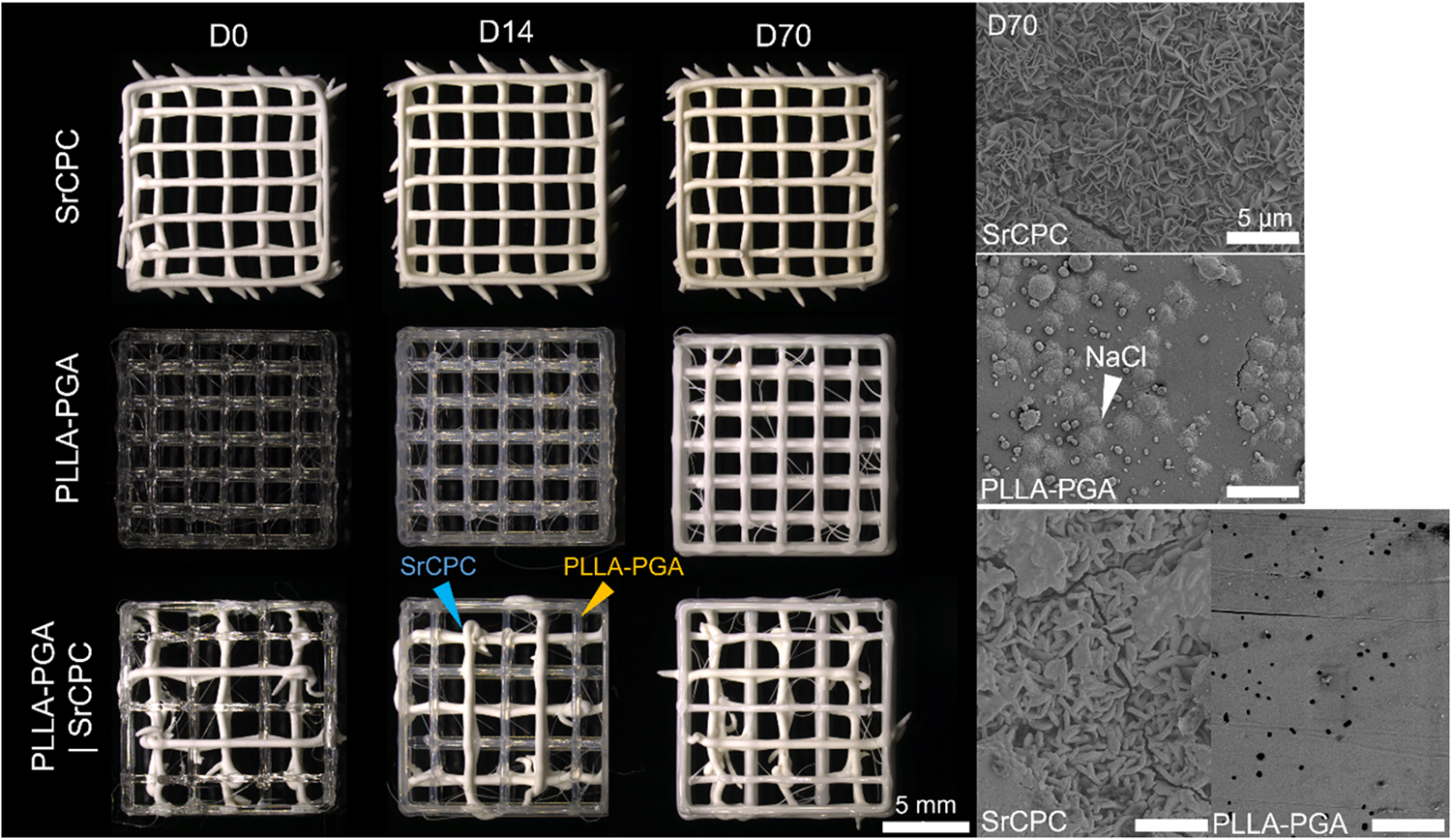
Microscopic images showing the degradation of all scaffold conditions in αMEM over a course of 70 days. Left: stereo light microscope; right: SEM images.

To evaluate the surface sediments on the scaffolds, EDX was conducted on gold-coated samples (Figure 5). SrCPC surfaces revealed elemental signals for C, O, Na, Mg, Sr, P, Cl, and Ca, with O, Ca, Sr, and P originating from the SrCPC. The presence of Na, Mg, and Cl likely reflects the deposition from the culture medium. Interestingly, after 70 days (D70), no Sr was detected on the surfaces of the biphasic scaffolds, and the monophasic SrCPC scaffolds exhibited a Sr peak similar to that on day 0 (D0).

**Figure 5:**
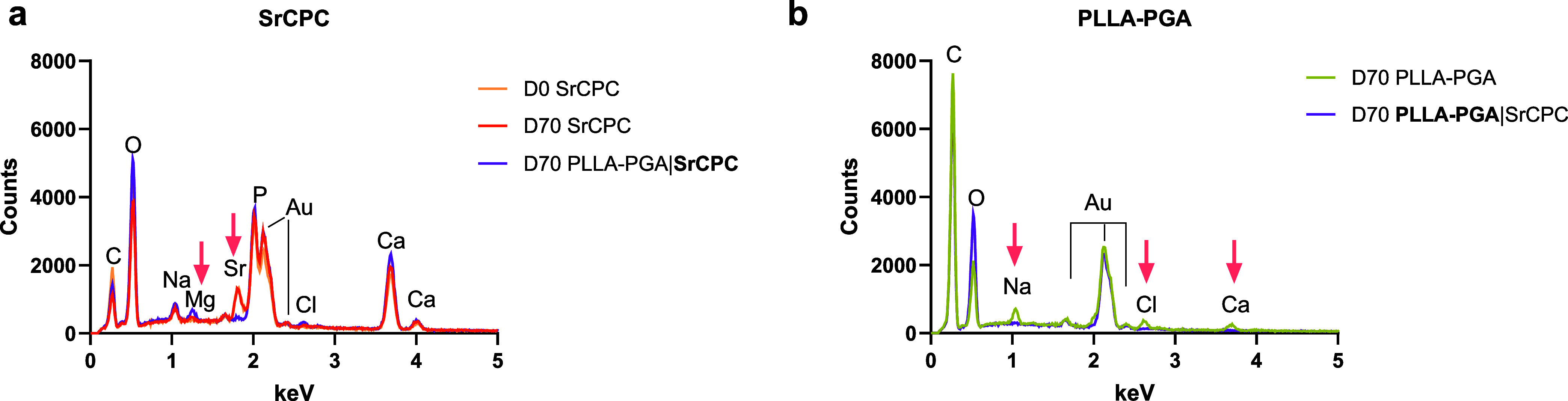
EDX measurements on selected spots of the surfaces of the monophasic and biphasic scaffolds for the SrCPC surfaces (a) and PLLA–PGA surfaces (b). Measurements of PLLA–PGA on D0 were not possible.

The PLLA–PGA surfaces posed challenges for EDX due to the high acceleration voltage (7 kV), which damaged the surface upon application. However, after 70 days of incubation, the surfaces primarily showed high C and O contents, as expected, but only the monophasic scaffolds exhibited signals for Na, Cl, and Ca, suggesting surface adsorption of NaCl and CaCl_2_.

Furthermore, the ion exchange dynamics of calcium, phosphate, and strontium between the culture medium and scaffolds were systematically evaluated over a 70-day period (Figure 6). As anticipated, the monophasic PLLA–PGA scaffolds exhibited negligible interactions with the tested ions, showing neither uptake nor release of calcium, phosphate, or strontium. In contrast, monophasic SrCPC scaffolds demonstrated continuous calcium uptake, accompanied by continuous release of both phosphate and strontium ions. For the biphasic condition, the calcium uptake decreased due to the lower amount of SrCPC in the scaffolds (Figure 6a,d). Interestingly, no phosphate was released (Figure 6b,e), and the Sr release decreased after 14 days of incubation (Figure 6c,f).

**Figure 6:**
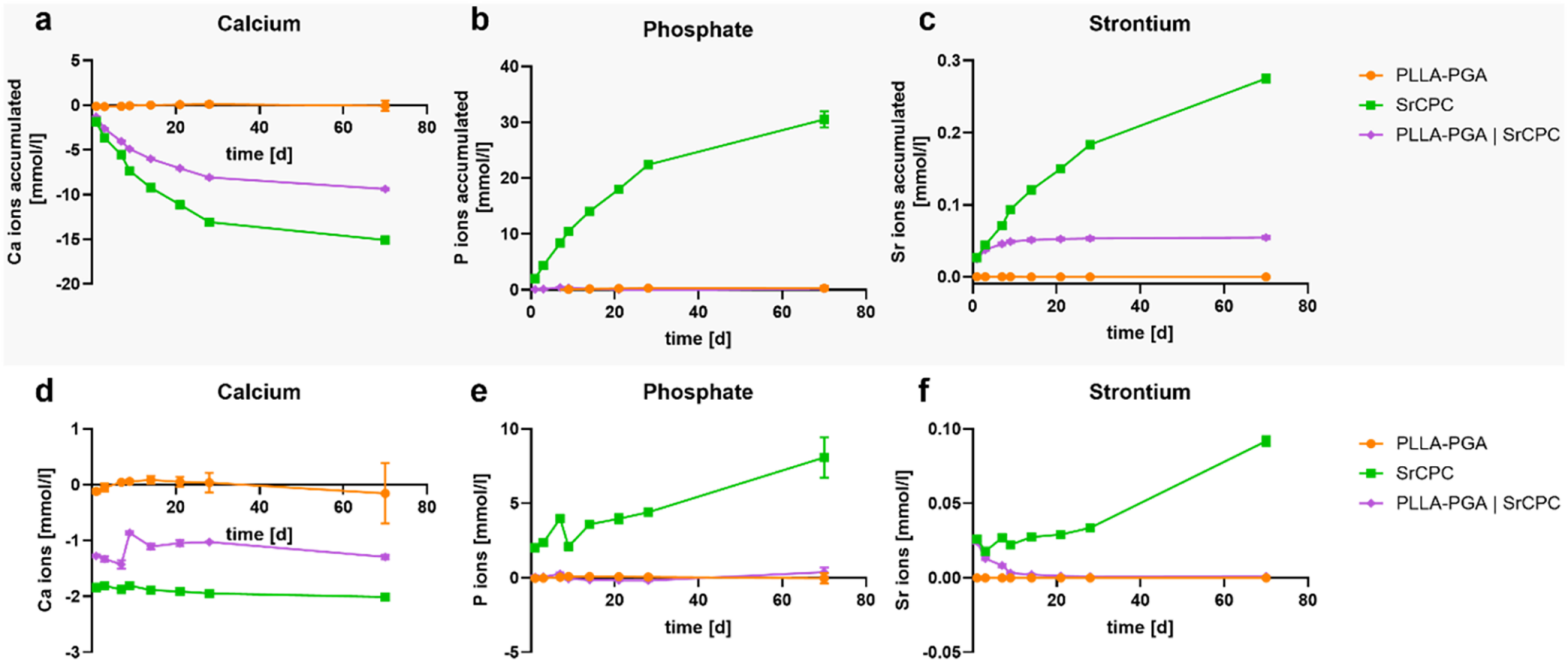
Ion release and uptake in the cell culture medium for calcium ((a, d), *n* = 3), phosphate ((b, e), *n* = 3), and strontium ((c, f), *n* = 3) over a cultivation period of 70 days; medium blank was subtracted; accumulated release curves ((a–c); positive slope indicates release, negative slope indicates uptake) and measured release within the supernatants ((d–f); positive values indicate release, negative values indicate uptake). Data points represent mean ± SD.

### 3.4 Fluorescence Microscopy and Biochemical Characterization of Cellular Response

The cytocompatibility of the biphasic scaffolds was evaluated by seeding SaOS-2 cells on printed and postprocessed PLLA–PGA/SrCPC scaffolds; monophasic scaffolds consisting of either SrCPC or PLLA–PGA served as references (Figure 7a). Fluorescence microscopic analysis revealed that 1 day after seeding with a high cell number (5 × 10^5^ cells/scaffold), all three scaffold types were homogeneously covered with cells (Figure 7b). Also, after 14 days of cultivation, cells were detected all over the scaffolds; however, in the biphasic condition, differences in the cell density between the SrCPC and the PLLA–PGA strands were observed: although the PLLA–PGA strands were completely covered with cells, the cell density on the SrCPC strands seemed to decrease (Figure 7b).

**Figure 7:**
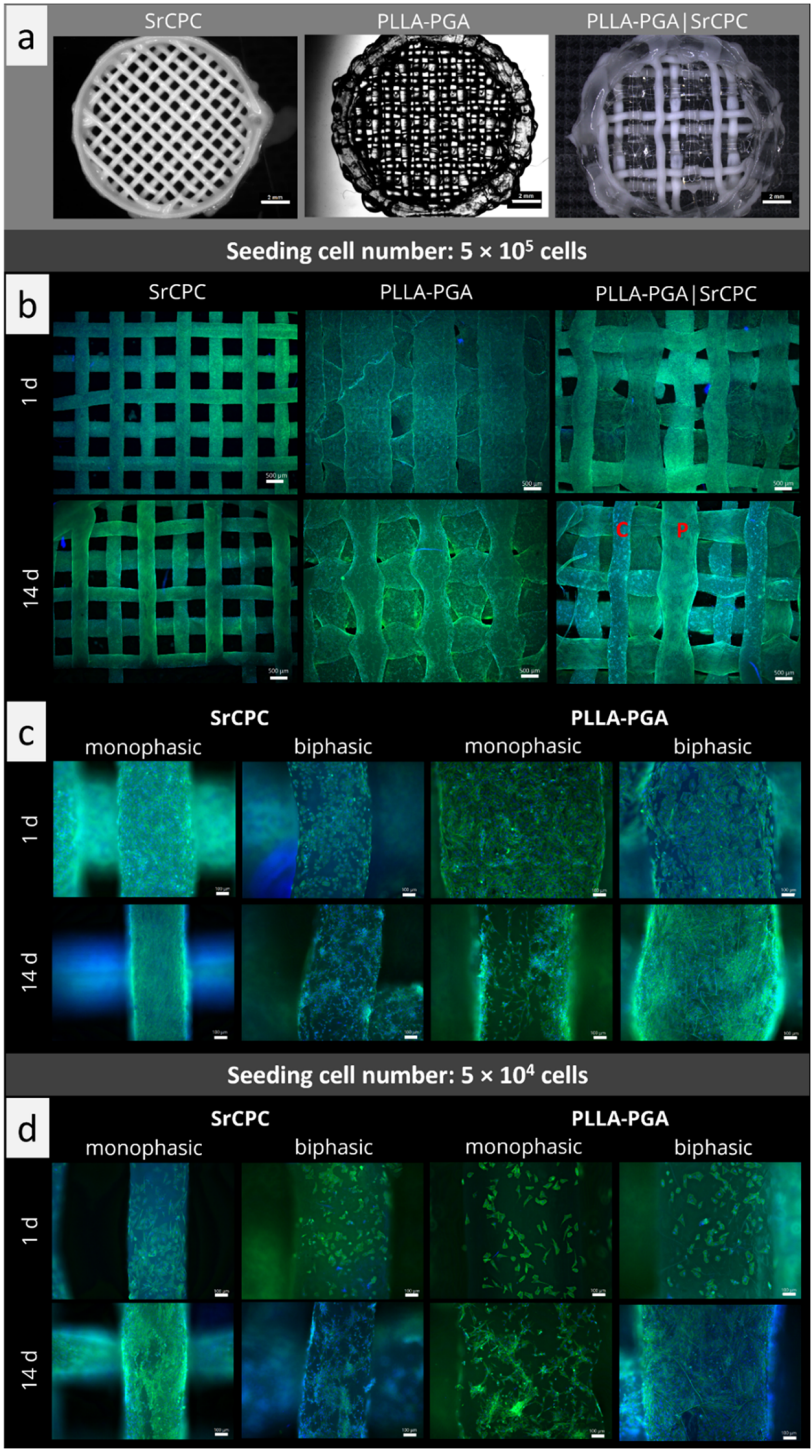
Monophasic SrCPC and PLLA–PGA scaffolds and biphasic PLLA–PGA/SrCPC scaffolds seeded with SAOS-2. Printed scaffolds used for cell culture experiments; scale bars = 2 mm (a). Overview fluorescence microscopic images of scaffolds seeded with 5 × 10^5^ cells/scaffold after 1 and 14 days of cultivation (b); adherent cells were stained with DAPI (cell nuclei; blue) and phalloidin (actin cytoskeletons; green), scale bars = 500 μm, C = cement (SrCPC), *P* = polymer (PLLA–PGA). Cell attachment and density on SrCPC and PLLA–PGA strands in monophasic and biphasic scaffolds seeded with 5 × 10^5^ cells/scaffold (c) or 5 × 10^4^ cells/scaffold (d). Detailed fluorescence microscopic images of scaffolds after 1 and 14 days of cultivation. Cells were stained with DAPI (cell nuclei; blue) and phalloidin (actin cytoskeletons; green). Scale bars = 100 μm.

A more detailed analysis of this phenomenon provides interesting insights, indicating the mutual influence of both materials. In Figure 7c, higher magnification images of single strands of SrCPC and PLLA–PGA, respectively, in the monophasic vs the biphasic scaffolds are shown after seeding with a high cell number (5 × 10^5^ cells/scaffold). One day after seeding, the SrCPC strands in the monophasic scaffolds were completely covered with cells, which were well spread. In contrast, SrCPC strands in the biphasic hybrid scaffolds were less densely covered with cells, which also exhibited a less spread and more rounded morphology. Cell adhesion to the PLLA–PGA strands was similar for the monophasic and biphasic scaffolds; cell density appeared to be slightly higher on the monophasic scaffolds. After 14 days of cultivation, the SrCPC strands showed the same picture; in the monophasic scaffolds, they were densely covered with spread cells, whereas in the biphasic scaffolds, the cell density barely increased compared to day 1, although the cells now exhibited a more spread morphology. Surprisingly, on day 14, a difference between mono- and biphasic scaffolds was observed for PLLA–PGA strands. Although the cell density in the monophasic scaffold seemed to decrease, it increased in the biphasic scaffolds. Conducting the experiment with a 10-fold lower seeding cell number (5 × 10^4^ cells/scaffold) indicated again an interaction of both materials within the biphasic scaffolds (Figure 7d): One day after seeding, the cell density and morphology on SrCPC and PLLA–PGA strands were similar between mono- and biphasic scaffolds; due to the lower seeding cell number, the cell density was significantly lower compared to the scaffolds shown in Figure 7c. On day 14, SrCPC strands in the monophasic scaffolds were densely colonized by cells, indicating strong cell proliferation, whereas they were not completely covered by the biphasic scaffolds. In contrast, the cell density on the PLLA–PGA strands increased strongly in the biphasic scaffolds from day 1 to day 14, resulting in dense colonization, whereas only a slight increase in the cell density was observed in the monophasic scaffold

Cell number quantification at different time points of cultivation (Figure 8a) largely reflected the results of the microscopic analysis. On day 1, the cell number on biphasic PLLA–PGA/SrCPC scaffolds was significantly lower than that on monophasic scaffolds. The significantly higher cell number on PLLA–PGA in comparison to the SrCPC scaffolds on day 1 could be a result of the increased polymer strand width (as visible in Figure 7). During further cultivation, the cell number decreased on the PLLA–PGA scaffolds, whereas the cell number increased on both the SrCPC and biphasic PLLA–PGA/SrCPC scaffolds. Consequently, on days 7, 14, and 21, the values were significantly lower on PLLA–PGA compared to SrCPC and PLLA–PGA/SrCPC scaffolds, and no significant differences were detected between SrCPC and PLLA–PGA/SrCPC scaffolds.

**Figure 8:**
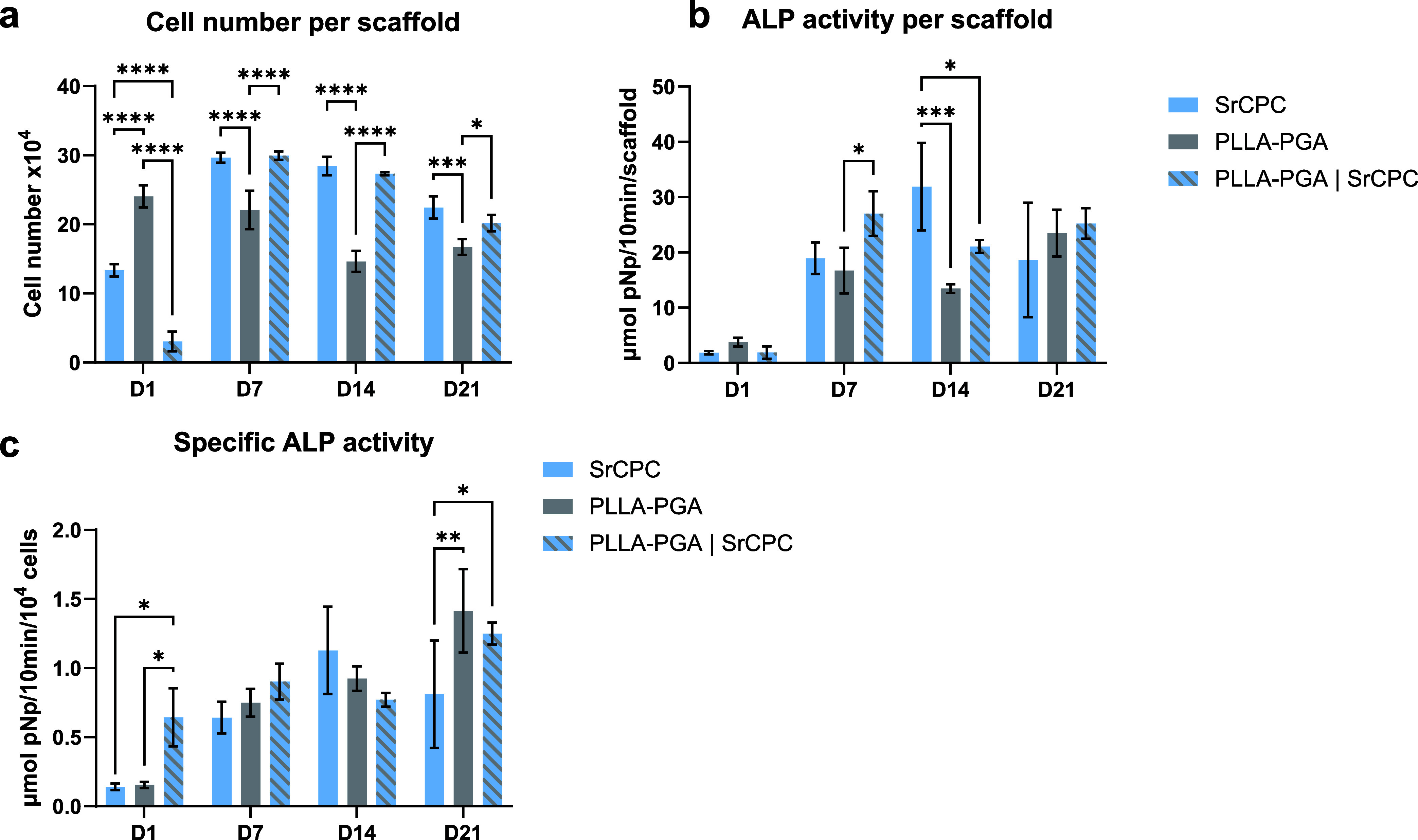
Cell growth and ALP activity of SAOS-2 cells cultured on monophasic SrCPC and PLLA–PGA scaffolds in comparison with biphasic PLLA–PGA/SrCPC scaffolds. Cell number was correlated with the cytosolic LDH activity measured after cell lysis (a), and the ALP activity as an indicator of osteogenic differentiation is shown as absolute activity per scaffold (b) and as specific activity in relation to the cell number (c) (*n* = 3, mean ± SD, *p < 0.05, **p < 0.01, ***p < 0.001, ****p < 0.0001).

The specific ALP activity (ALP activity per cell number) as an early marker of osteogenic differentiation increased on all three scaffold types over the cultivation time (Figure 8b). The significantly higher values on PLLA–PGA and biphasic PLLA–PGA/SrCPC scaffolds on day 21 could be a result of the delayed differentiation of the cells attached to the polymer strands, as the maximum ALP activity on SrCPC scaffolds was already on day 14. The ALP activity per scaffold, reflecting the sum of the cell number and specific ALP activity, was significantly increased on the biphasic PLLA–PGA/SrCPC compared to the PLLA–PGA scaffolds on day 7; on day 14, the values were significantly highest on SrCPC and lowest on PLLA–PGA scaffolds (Figure 8c).

## 4 Discussion

In this study, we established a printing and postprocessing protocol for fabricating biphasic scaffolds composed of SrCPC and PLLA–PGA, aiming to mitigate the acidic degradation of PLLA–PGA and thereby enhance the biocompatibility and bioactivity of the resulting implants. As anticipated, the biphasic constructs exhibited a combination of material-specific characteristics of both components. In addition, we observed a mutual influence between the two materials in *in vitro* experiments.

### 4.1 Printing and Postprocessing

The fabrication of biphasic PLLA–PGA/SrCPC scaffolds presents specific challenges arising from the combination of fundamentally different material classes and processing temperatures in extrusion-based printing. Monophasic scaffolds were successfully produced following the established procedures.^26,34^ The disparity in processing conditions became obvious for biphasic printing and aggravated the printing process. Particularly, the disparity between the high-temperature extrusion of PLLA–PGA and the ambient-temperature extrusion of SrCPC pastes resulted in limited strand adhesion at the material interface, adversely affecting the geometric accuracy. This challenge was particularly pronounced when molten PLLA–PGA was deposited onto SrCPC strands, making pure SrCPC and biphasic frames unfeasible for scaffold stabilization. Thus, the only viable option was to print a supportive PLLA–PGA frame to maintain the structural integrity. Moreover, the PLLA–PGA strands exhibited poor self-adhesion, likely resulting from rapid solidification induced by substantial temperature gradients between the nozzle output and ambient conditions. Consequently, the strands might have solidified prematurely, diminishing the interface quality. Although raising ambient temperatures could theoretically enhance strand adhesion,^35^ such adjustments were not feasible within our printer’s setup. An alternative attempt employing the Arburg Plastic Freeforming 3D printing system,^36,37^ designed for thermoplastic extrusion at elevated ambient temperatures (55 °C), was incompatible with the SrCPC extrusion. As a compromise, the printing temperature in the BioScaffolder system was raised to 170 °C. Therefore, we aimed to balance improved strand adhesion and strand geometry retention while minimizing the risks of thermal degradation.^35,38^

Reduced printing speeds further improved the adhesion between the different phases, consistent with previous composite fabrication strategies.^39^ Despite these adjustments, slight variations in the strand width were observed, particularly for the strand diameters between the adhesion points (Figure 2k). In contrast to reports on composite systems combining polymer phases with ceramic fillers,^40,41^ our approach did not exhibit thermal expansion immediately postextrusion.

Overall, biphasic printing of thermoplastics and extrudable ceramic pastes like PLLA–PGA/SrCPC offers a more flexible method to integrate mineral phases into polymeric scaffolds and to tailor the polymer/ceramic ratio over a broader range compared to 3D printing of composite materials, in which ceramic fillers are integrated into the polymer phases.^26^ However, the process still highlights the general difficulty of combining thermoplastic and ceramic phases within an extrusion-based approach.

In terms of postprocessing, our protocol—comprising a 3-day setting phase under humid conditions, subsequent gentle washing in 0.9% NaCl to remove the SrCPC carrier liquid, and final sterilization via γ-irradiation (max. 25 kGy)—was specifically adapted to maintain both cement integrity and polymer stability while aiming for clinical applicability. Nevertheless, the NaCl treatment might have resulted in higher oil residue levels compared to an acetone treatment as performed for monophasic 3D printed CPC implants.^17,31^ However, previous studies have demonstrated that residual oil does not negatively influence cellular behavior;^16^ all components of the carrier liquid are biocompatible, and the CPC paste is approved for clinical application as an injectable bone defect filling material.^42,43^

### 4.2 Degradation in Water

As intended, the SrCPC strands effectively moderated the pH drop typically associated with the hydrolytic degradation of PLLA–PGA in aqueous environments.^44^ The SrCPC component acted as a buffering phase, delaying and attenuating the acidification of the surrounding medium. Surprisingly, in comparison to our previous study, in which composite scaffolds of PLLA–PGA and various mineral microparticles were investigated,^26^ a significantly earlier pH drop for both monophasic PLLA–PGA and biphasic scaffolds was observed in the present work (14 days vs 84 days). This accelerated acidification emphasizes the thermal degradation of the polymer due to the fabrication process.^45^ Furthermore, ceramic fillers like SrHAp, which was obtained from set SrCPC, buffered the previous system over the entire 112-day study period,^26^ whereas in this study, an evident pH drop occurred after 98 days (14 weeks). Nevertheless, SrCPC buffered the pH effectively to approximately 4.4, substantially higher than the pH of around 2.5 observed for monophasic scaffolds but consistent with other studies.^34^ In direct comparison with Ahlfeld et al.,^26^ these findings underline the substantial influence of fabrication techniques on scaffold degradation. Specifically, the Arburg Freeformer system utilized in the previous study may have induced less thermal degradation compared to the BioScaffolder employed in the present study (Supporting Information: Figure S1). Decisively, the results of the present study demonstrate that a buffering effect can also be achieved if the mineral phase is not in direct contact with the polymer, as is the case with composites, but is in the immediate vicinity.

In concurrence with the pH drop, the conductivity of the supernatant continuously increased for monophasic PLLA–PGA scaffolds, supporting the early onset and continuous degradation of PLLA–PGA. The monophasic SrCPC scaffold showed a constant conductivity, which is in accordance with the constant ion release known from previous studies.^15,34^ Interestingly, the conductivity in the supernatants of the biphasic PLLA–PGA/SrCPC scaffolds was lower than that of both monophasic scaffold types until week 12. This can be explained by the lower ion release from the SrCPC component due to its lower number of strands and the decelerated degradation of PLLA–PGA when compared with that of the monophasic scaffolds. The latter could be caused by the buffering of the acidic degradation products, which are known to accelerate the hydrolysis of ester bonds autocatalytically.^46,47^ The sharp increase in conductivity in the biphasic PLLA–PGA/SrCPC group after week 12 can be linked to the decrease in pH, indicating an increasing degradation of the PLLA–PGA component. Simultaneously, the pH drop to approximately 4.4 can lead to local dissolution of the hydroxyapatite^48^ in the SrCPC strands, and thus, an increased ion release within the biphasic scaffolds contributes to a higher conductivity, which exceeded that of the two monophasic groups at later time points.

Over the course of 24 weeks, all scaffold types exhibited a decline in Young’s modulus and compressive strength, consistent with degradation-related mechanical weakening. As expected, monophasic SrCPC scaffolds maintained their mechanical properties during the first 4 weeks, with only minor reductions observed after 24 weeks, correlating with their nearly unchanged structural integrity (Figure 3e). Monophasic PLLA–PGA scaffolds initially displayed favorable mechanical properties but showed a steep decline over time, corroborating previous findings,^37^ and underscoring the pronounced degradation of the polymer, as further evidenced by partial scaffold fractures during the degradation study (Figure 3e).

Biphasic scaffolds, despite containing a stabilizing PLLA–PGA frame, more closely resembled the mechanical behavior of monophasic SrCPC, emphasizing that the most brittle phase within the architecture governed the overall mechanical performance of the given scaffold architecture. Notably, the compressive strength of the biphasic scaffolds showed a slight increase compared to that of the monophasic SrCPC within the first 4 weeks, suggesting that the PLLA–PGA frame initially contributed to load-bearing. However, after 24 weeks, the biphasic scaffolds displayed markedly reduced mechanical integrity, with substantial material loss (Figure 3e). This observation aligns with extensive polymer degradation and supports the hypothesis that local pH shifts induced by polymer hydrolysis may have further compromised SrCPC by promoting its dissolution and weakening the interfacial bonding between phases. The latter will not occur after implantation *in vivo*; it can be attributed to the artificial test condition, that is, in water without any buffering component, which was chosen to characterize the degradation of the polymer component according to the DIN EN ISO 10993-13 and to monitor the mutual influence of both materials in the absence of further influencing factors.

This behavior diverges from the findings for composite systems, where ceramic fillers are dispersed within polymer matrices. In such cases, the ceramic addition often enhances the mechanical performance.^3,40,41,49^ However, mechanical reinforcement seems to depend on the ceramic content: for instance, PCL/SrHAp composites showed increased mechanical strength up to 30% ceramic loading, followed by a sharp decline at 50%.^39^ Similarly, Akindoyo et al. reported reduced tensile and impact strengths with increasing hydroxyapatite (HAp) content in PLA/HAp composites,^50^ while Shuai et al. found that increasing the PGA and HAp contents in HAp/PLLA–PGA systems led to decreased mechanical stability.^51^ Bayart et al. likewise observed that 90% PLA blended with 10% HAp exhibited a lower Young’s modulus and compressive strength than monophasic PLA, a trend that intensified after five months of degradation.^37^

On the other hand, the dispersion of ceramic fillers in a polymer matrix can have significant disadvantages. Besides a changed melting behavior, which results in a smaller time window for processing and a limited range of applicable printing nozzles, composites of 90% PLLA–PGA and 10% ceramic filler particles (SrHAp and others) exhibited considerable swelling when incubated in cell culture medium or simulated body fluid.^26^

These findings collectively underscore the fundamental distinction between the integration of ceramic phases within a polymer matrix and their printing as discrete phases.

DSC analysis revealed pronounced thermal changes in PLLA–PGA for both monophasic and biphasic scaffolds over a 24-week degradation period in water. These alterations are closely associated with the fabrication methods. Scaffolds printed using an Arburg Freeformer system and subjected to the same degradation protocol exhibited comparatively less pronounced reductions in the glass transition temperature (*T*_g_: 61 to 49 °C) and melting temperature (*T*_m_: 164 to 151 °C), as illustrated in Supporting Information: Figure S1.

Notably, a double melting peak was observed in both fabrication techniques during the first 4 weeks and persisted in the Freeformer-printed scaffolds after 24 weeks. This phenomenon is commonly attributed to the presence of imperfect or metastable α-crystals formed during the crystallization of PLLA, which undergo melting, structural reorganization, and recrystallization during heating. The lower melting temperature peak (Peak I) corresponds to the melting of primary α-crystals, while the higher-temperature peak (Peak II) is attributed to the melting of recrystallized, more thermally stable α-crystals. Both crystal phases are formed during the fabrication process and reflect the typical thermal behavior of PLLA, consistent with previous studies on its crystallization and melting characteristics.^52–55^ Additionally, the disappearance of exothermic peaks in scaffolds printed with the BioScaffolder after 24 weeks suggests advanced structural reorganization of the polymer matrix, including another recrystallization process.

In contrast, the biphasic scaffolds exhibited a single melting peak throughout the degradation study, indicating the predominant formation of thermally stable α-crystals in PLLA. As observed for monophasic scaffolds, the progressive reduction in *T*_g_ can be attributed to the preferential degradation of the amorphous domains, resulting in an increased proportion of crystalline regions. Notably, the decrease in *T*_g_ was more pronounced in scaffolds printed with the BioScaffolder compared to those produced with the Freeformer. This difference likely stems from the higher ambient temperature during Freeformer processing, which reduces the cooling rate and promotes more extensive crystallization during fabrication.^41,56,57^ Consequently, the lower amorphous content in Freeformer-printed scaffolds leads to reduced hydrolytic susceptibility and slower overall degradation.

Consistent with previous studies, the incorporation of ceramic content resulted in the absence of exothermic peaks in the DSC thermograms.^34,58^ Nevertheless, the observed reduction in *T*_g_ suggests degradation behavior comparable to that of monophasic PLLA–PGA scaffolds. The absence of significant attenuation of exothermic signals may be attributed to a less pronounced recrystallization process or to limitations inherent to the DSC measurement conditions, as also observed in the monophasic SrCPC controls.

### 4.3 Degradation in Cell Culture Medium and Cellular Response of Osteoblastic Cells

Although the degradation study in water provided insightful results regarding the pH development and degradation behavior of biphasic scaffolds in comparison to the monophasic PLLA–PGA scaffolds, it did not contribute to understanding the observed cell behavior; therefore, an additional degradation study in cell culture medium with a special focus on bioactivity and ion release/uptake was conducted. The degradation in the medium caused a similar whitening of the PLLA–PGA strands as the degradation in water. In both cases, this effect indicates the formation of microcracks and pits, which facilitate increased fluid diffusion and, consequently, accelerate degradation. This process also alters light scattering, thereby changing the material’s color. Upon immersion in water, autocatalytic degradation may be promoted due to the pronounced pH shift.^59^ In contrast, immersion in a buffered medium is more likely to favor hydrolytic degradation.^33,60^

After 70 days of incubation in cell culture medium, we detected monophasic SrCPC, the nanocrystalline structures known for calcium phosphate cements.^14^ For monophasic PLLA–PGA, most likely NaCl and CaCl_2_ from the medium adsorbed on the surfaces, form a continuous layer similar to our previous study.^26^ For biphasic scaffolds, SEM analysis revealed clean yet perforated PLLA–PGA strand surfaces (Figure 4), consistent with previous PLLA–PGA degradation studies.^61,62^ This underscores that precipitates seen on monophasic PLLA–PGA scaffolds preferentially attach to SrCPC rather than to PLLA–PGA surfaces. The perforations further reflect pronounced PLLA–PGA degradation over the 70-day period. The SrCPC phase in biphasic scaffolds showed a rounded crystalline structure with flat patches, suggesting attachment of oligomers from the degrading polymer.^60^

Most SEM observations were corroborated by the EDX measurements (Figure 5). For monophasic SrCPC, the expected elements were confirmed, with a distinct Sr phase visible on the surfaces on day 0 and after 70 days of incubation in the cell culture medium. In biphasic scaffolds, however, no Sr phase was detectable on SrCPC surfaces after 70 days of incubation, correlating with the reduced Sr release observed after 20 days in the ion release analysis. This suggests that the precipitates formed on the SrCPC strands (Figure 4) produced a shielding effect, thereby attenuating Sr release. EDX further revealed Mg adsorption on SrCPC surfaces in biphasic scaffolds, indicating that Mg from the medium may have reacted with phosphate to form a continuous MgP layer. However, MgP formation under the given conditions appears questionable, as alkaline pH values (>8.6) are generally required.^63^ In addition, it is unclear why no Mg adsorption was detected on the SrCPC surfaces in monophasic scaffolds.

An additional complexity is the complete absence of phosphate ion release from the SrCPC in the biphasic scaffolds, evident from the very start of the degradation study. Monophasic SrCPC scaffolds showed phosphate release, which is typical for CPC.^18^ Again, the reason for this observation is unclear. Although MgP formation could partly account for it, the assumption that, at every time point, all phosphate released from the cement directly reacts with Mg ions—without any detectable fluctuations in phosphate concentration in the medium, as implied by the release profile in Figure 6b,e (after subtraction of the phosphate baseline of the medium)—appears unrealistic. The alternative hypothesis that phosphate ions are not released from SrCPC in the presence of PLLA–PGA in biphasic scaffolds, partially fitting to the decrease in Sr release to 0 mmol/L within the first 20 days, also seems questionable. Although we cannot fully explain our observations, we can clearly state that in the biphasic condition, both materials have an influence on each other and that the hydrolysis rate of solid polyesters depends on the local ionic environment, pH, oligomer diffusivity, and matrix porosity.^60^ To the best of our knowledge, such a combination of effects has not been reported previously and contrasts markedly with the results from thermoplastic–ceramic composites, such as the PLLA–PGA + SrHAp composite described by our group previously.^26^ Further investigations should include continuous pH monitoring, even in buffered systems, to detect possible local alkaline and/or acidic shifts,^64^ followed by X-ray diffractometry to more precisely characterize material surface transformations.

Aging in the medium induced distinct superficial layers on monophasic PLLA–PGA and biphasic SrCPC surfaces, which altered local fluid transport, ion exchange, and degradation. Consequently, the altered material properties affected the cell colonization with the human osteosarcoma cell line SAOS-2, which is a standardized cell line often used for cytocompatibility analyses.^65^ Although monophasic SrCPC supported dense, well-spread cell layers, as described previously,^15^ the same ceramic phase in biphasic scaffolds showed impaired cell adhesion and proliferation (Figure 7c,d). This suggests that the local surface environment or ionic microclimate, potentially altered by adjacent polymer degradation, may impede cell adhesion to SrCPC. Again, whether the formation of MgP on the SrCPC surface—depending on its concentration, struvite is potentially cytotoxic^66^—has contributed to this phenomenon is questionable. In contrast, for PLLA–PGA strands, the picture was the opposite: After an initial cell attachment (day 1), which was comparable between monophasic and biphasic scaffolds, the cell density did not significantly increase (5 × 10^4^ group) or even decrease (5 × 10^5^ group) over 14 days of cultivation on monophasic PLLA–PGA scaffolds, whereas the cell density strongly increased on PLLA–PGA strands in the biphasic scaffolds (in both, 5 × 10^4^ and 5 × 10^5^ group; Figure 7c,d). These observations indicate that the presence of SrCPC promoted cell proliferation, for example, via the release of Sr^2+^ ions, as observed in this early phase (Figure 6f) or by balancing local pH shifts caused by the onset of polymer degradation.

The activity of ALP, an early marker of osteogenic differentiation, peaked earlier in the monophasic SrCPC scaffolds (day 14), indicating that SrCPC supported osteogenic differentiation. In contrast, ALP activity on the PLLA–PGA and biphasic scaffolds continued to increase, reaching the highest values on day 21. Overall, the proliferation and the ALP activity on biphasic scaffolds were close to those on the monophasic SrCPC scaffolds and were often significantly higher than those on the monophasic PLLA–PGA scaffolds (Figure 8), highlighting the positive effect of combining SrCPC with PLLA–PGA.

### 4.4 Limitations of the Study and Outlook

The approach of combining SrCPC as a bioactive ceramic phase with PLLA–PGA as a biodegradable polymer by applying hybrid extrusion printing increases the complexity of the fabrication process, which encompasses both the printing and postprocessing procedures. When considering its application, it is important to carefully weigh whether the benefits of biphasic scaffolds justify the higher number of process steps and parameters. The in vitro investigations in the present study have shown that our first hypothesis was true, as a reduced pH drop during polymer degradation due to the buffering effect of the mineral phase was proven. However, the second hypothesis was only partially correct: although the cell experiments demonstrated significantly enhanced cell proliferation and alkaline phosphatase activity in the biphasic scaffolds compared to the monophasic PLLA–PGA scaffolds, the release of osteostimulatory strontium ions was suppressed in the long term by the presence of the polymer phase in the biphasic scaffolds. The superiority of hybrid scaffolds should also be investigated in a relevant bone defect model. This would also help assess the impact of local heterogeneity in cell–material interactions, observed within the biphasic scaffolds, on tissue ingrowth and new bone formation. From a mechanical perspective, nondegraded PLLA–PGA scaffolds fell within the physiologically relevant range of native bone,^67–69^ yet the SrCPC structures alone were inherently brittle, and the integration of the polymer phase did not sufficiently counteract this brittleness in the biphasic constructs. The mechanical properties declined over the 24-week observation period as intended, but specific in vivo degradation kinetics remain to be investigated.

## 5 Conclusions

We successfully established biphasic printing of thermoplastic polymers and ceramic pastes, although the printing parameters for PLLA–PGA required further optimization to minimize thermal damage. The main material properties were preserved, and the mineral phase buffered the acidity during polymer degradation, resulting in reduced acidification and improved biocompatibility. Degradation studies revealed medium- and composition-specific effects: in water, the PLLA–PGA component in biphasic scaffolds appeared to shift from the autocatalytic degradation typical of monophasic PLLA–PGA toward hydrolytic degradation, likely due to the buffering effect of SrCPC. In medium, degradation seemed to be mainly hydrolytic across all scaffolds and was accompanied by ion–surface interactions that produced distinct mineral deposits, potentially NaCl-rich layers on monophasic PLLA–PGA, hydroxyapatite on monophasic SrCPC, and a previously unreported magnesium-rich phase on SrCPC strands in biphasic constructs. The latter coincided with suppressed Sr release and an unclear absence of the phosphate release. These material-driven surface modifications directly affected cell colonization, with biphasic constructs more closely resembling the cellular response to monophasic SrCPC and showing enhanced proliferation and osteogenic activity compared with monophasic PLLA–PGA in our initial cell experiments. Collectively, these findings highlight the complexity of degradation where hydrolysis, ion exchange, oligomer diffusion, and pH-dependent buffering occur simultaneously. Nonetheless, biphasic printing has emerged as a promising strategy to tailor degradation and bioactivity in bone scaffolds, providing a broad foundation for future *in vivo* studies to clarify ion release mechanisms and their role in osteostimulation.

## Supplementary Material



## Notes

Differential scanning calorimetry (DSC) thermograms of PLLA–PGA scaffolds fabricated either with a Freeformer system or a GeSiM BioScaffolder, shown after incubation in water for 0, 2, 4, and 24 weeks (PDF)

## ⊥

Department of Oral, Maxillofacial, and Plastic Surgery, Rostock University Medical Center, Schillingallee 35, Rostock 18057, Germany
