# Supplementary material for: Biphasic Bone Implants through Hybrid Extrusion Printing of Thermoplastic Poly(lactic-co-glycolic) acid and Strontium-Modified Calcium Phosphate Bone Cement
Source: ACS Omega. 2026 Apr 30;11(18):26479–94. doi: 10.1021/acsomega.5c12496 (PMC13177265; doi:10.1021/acsomega.5c12496)
Supplement: Supplementary file 1 [file ao5c12496_si_001.pdf]

Biphasic bone implants through hybrid extrusion printing of thermoplastic poly(lactic-co-glycolic) acid and a strontium-modified calcium phosphate bone cement

*Max von Witzleben[1], Tilman Ahlfeld[1], Richard Frank Richter[1], Anna-Maria Placht[1], Constantin Greil[1], Corina Vater[1], Andreas Hoess[2], Sascha Heinemann[2], Stefanie Grom[3], Tatjana Fecht[3], Jan Marc Schehl[3], Tobias Wolfram[3], Frank Reinauer[3], Christian Bräuer[4], Günter Lauer[5], Michael Gelinsky[1], Anja Lode[1]**

[1]Centre for Translational Bone, Joint and Soft Tissue Research, University Hospital Carl Gustav Carus and Faculty of Medicine at Technische Universität Dresden, 01307 Dresden, Fetscherstr. 74, Germany

[2]INNOTERE GmbH, 01445 Radebeul, Meissner Str. 191, Germany

[3]KLS Martin SE & Co. KG, 78570 Mühlheim, Kolbinger Str. 10, Germany

[4]Department of Oral, Maxillofacial and Plastic Surgery, Rostock University Medical Center, 18057 Rostock, Schillingallee 35, Germany

[5]Department of Oral and Maxillofacial Surgery, University Hospital Carl Gustav Carus at Technische Universität Dresden, 01307 Dresden, Fetscherstr. 74, Germany

SUPPORTING INFORMATION

Differential Scanning calorimetry (DSC)

DSC was performed on PLLA-PGA scaffolds after incubation in water for 0, 2, 4, and 24 weeks to assess thermal stability over time. Scaffolds were fabricated using a Freeformer system (Arburg, Lossburg, Germany) under ambient conditions of 55 °C and 30 MPa pressure. The porous architecture was based on the design reported by Ahlfeld et al. (2023, https://doi.org/10.1039/D2BM02071H), with a strand thickness of 1 mm and pore dimensions of 1.3 mm (x) × 0.6 mm (y), yielding a diagonal pore size of approximately 1.4 mm.

Over the first 4 weeks, the thermal properties of the PLLA-PGA copolymer remained stable. However, after 24 weeks of aqueous incubation, DSC analysis revealed significant thermal degradation, as evidenced by a reduction in the melting and glass transition temperatures from 164 °C to 151 °C and from 61 °C to 49 °C, respectively.

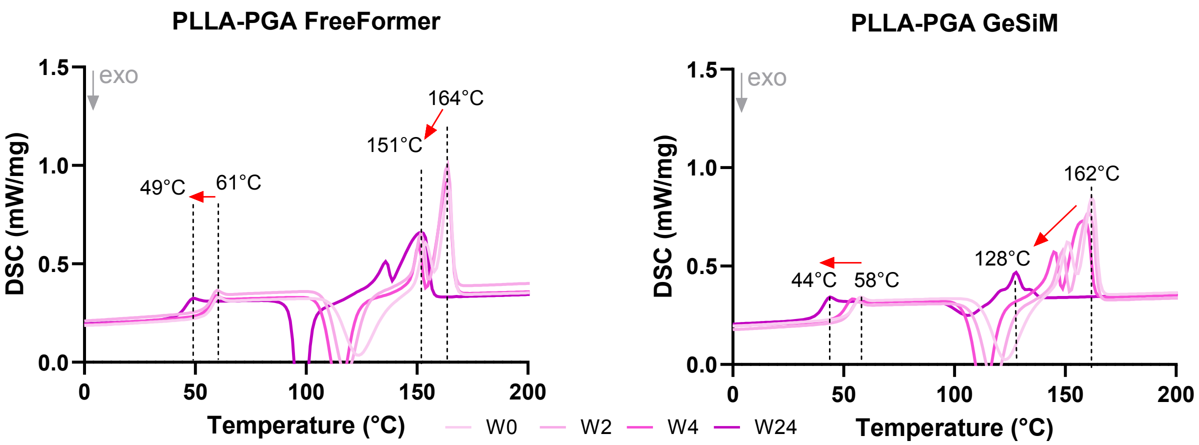

Figure S1. Differential scanning calorimetry (DSC) thermograms of PLLA-PGA scaffolds fabricated either with a Freeformer system or a GeSiM BioScaffolder, shown after incubation in water for 0, 2, 4, and 24 weeks.
